# Enhancing the evaluation of acute ischemic stroke risk in individuals with non-valvular atrial fibrillation by including laboratory indicators

**DOI:** 10.1038/s41598-024-57497-x

**Published:** 2024-03-21

**Authors:** Xin Hou, Xiaohong Zhang

**Affiliations:** Department of Cardiology, The Third Affiliated Hospital of Anhui Medical University (The First People’s Hospital of Hefei), Hefei, 230001 Anhui China

**Keywords:** Non-valvular atrial fibrillation_1_, Lipoprotein (a)_2_, Red blood cell distribution width_3_, Acute ischemic stroke_4_, Neuroscience, Medical research

## Abstract

To investigate the clinical significance of the CHA_2_DS_2_-VASc-60 score, lipoprotein (a) [Lp(a)], red blood cell distribution width (RDW), and their combined effect in patients with non-valvular atrial fibrillation (NVAF) who experience acute ischemic stroke (AIS). This retrospective analysis was conducted on the clinical data of hospitalized patients with NVAF at the Third Affiliated Hospital of Anhui Medical University between April 1, 2020, and April 1, 2023. Based on the diagnosis of acute ischemic stroke (AIS), the patients were divided into two groups: the AIS group (150 cases of NVAF patients with comorbid AIS) and the non-AIS group (163 cases of NVAF patients without AIS). We performed CHA_2_DS_2_-VASc-60 scoring for all patients and collected their laboratory indicators and echocardiographic indicators during hospitalization. The study comprised 313 individuals with NVAF in total. There is a statistically significant difference (*P* < 0.05) in the comparison of CHA_2_DS_2_-VASc-60 score (5.68 ± 1.12 vs. 3.67 ± 1.47), Lp(a) [23.98 (13.28, 42.22) vs. 14.32 (7.96, 21.91)] and RDW (13.67 ± 1.25 vs. 12.94 ± 0.76) between NVAF patients with and without concomitant AIS. The results of the Spearman correlation analysis demonstrate a positive association between Lp(a) and RDW levels and both the CHA_2_DS_2_-VASc score and the CHA_2_DS_2_-VASc-60 score in patients with NVAF. Multivariate logistic regression analysis revealed that CHA_2_DS_2_-VASc-60 score [*OR* = 6.549, 95% *CI*: 4.110–10.433, *P* < 0.05], Lp(a) [*OR* = 1.023, 95% *CI*: 1.005–1.041, *P* < 0.05], and RDW [*OR* = 1.644, 95% *CI*: 1.071–2.525, *P* < 0.05] were independent risk factors for AIS in patients with non-valvular atrial fibrillation (NVAF). The receiver operator characteristic (ROC) curves showed that the area under the curve of CHA_2_DS_2_-VASc-60 score, Lp(a), RDW, and CHA_2_DS_2_-VASc-60 score combined with Lp(a) and RDW predicted that NVAF patients with AIS were 0.881 [95% *CI*: 0.804–0.906], 0.685 [95% *CI*: 0.626–0.744], 0.695 [95% *CI*: 0.637–0.754], and 0.906 [95% *CI*: 0.845–0.921], respectively. The CHA_2_DS_2_-VASc-60 score, Lp(a), and RDW were significantly increased in NVAF patients with AIS, which were independent risk factors for NVAF patients with AIS. The combination of the three has a high predictive capacity for NVAF patients with AIS.

## Introduction

Atrial fibrillation (AF) has emerged as a significant global health issue, as its incidence and prevalence have increased in tandem with the aging of the population^1^. According to statistics, the global population with atrial fibrillation is approximately 50 million in 2020^2^. The risk of ischemic stroke (IS) is considerably increased by a factor of 5–6 in individuals with atrial fibrillation (AF)^3^; there are reports of high incidence and mortality rates associated with ischemic stroke caused by AF. According to the survey, among patients with atrial fibrillation in China, the overall prevalence of ischemic stroke is 24.8%, leading to a mortality rate of nearly 20%^4^. Atrial fibrillation raises the risk of ischemic stroke by 1.92% per year. The condition may result in a mortality rate of around 20% and a disability rate of over 60%^5^.

Considering the prethrombotic state in which atrial fibrillation exists, the primary focus of patient management is the prevention of thromboembolism^6^. In patients with AF, the CHA_2_DS_2_-VASc scoring system (hypertension, heart failure, diabetes, vascular disease, age 65–74 years, and female gender each received one point, while age over 75 years and a history of stroke, transient ischemic attack, or thromboembolism received two points, respectively) is a valuable clinical tool for assessing the risk of stroke. The ability of the CHA_2_DS_2_-VASc scoring system to distinguish between individuals with atrial fibrillation who were really at low risk was shown in a large 2010 study (the c-statistic was 0.606)^7^. Consequently, a number of guidelines advise using the CHA_2_DS_2_-VASc scoring system to evaluate the risk of embolism in individuals suffering from atrial fibrillation^8^. The simplicity and practicability of the CHA_2_DS_2_-VASc scoring system are its principal advantages. Nevertheless, its predictive efficacy for stroke or other cerebrovascular ischemic events is considered to be suboptimal (particular patient populations, including those with renal insufficiency or of Asian descent) in real-world cohorts^9,10^. Several studies have attempted to develop new scoring systems to enhance the predictive ability of stroke risk. Cha et al. developed a new stratified system, CHA_2_DS_2_-VAK, which omits the female gender category and adds CKD. The AUC of the CHA_2_DS_2_-VASc in the cohort was 0.639 (95% CI 0.62–0.65, P < 0.001) by ROC curve. The new CHA_2_DS_2_-VAK system had an AUC of 0.650 (95% CI 0.64–0.66, P < 0.001)^11^. In recent years, the use of the CHA_2_DS_2_-VASc-60 score has been recommended in China for assessing stroke risk in patients with atrial fibrillation who are Asian and have a reduced age threshold for increased stroke risk. The scoring system adjusts the age to receive 1 point for individuals aged 60–64 and 2 points for those aged 65 and above.

Biomarkers have become highly effective instruments for diagnosing, predicting the prognosis, and providing therapeutic guidance for a wide range of cardiovascular conditions. They facilitate early detection, risk stratification, and prognosis^12,13^. An improved understanding of established and emergent molecular mechanisms and biomarkers could enhance the ability to identify patients at risk for ischemic stroke with NVAF and facilitate the provision of anticoagulation guidance^14^. Previous studies have demonstrated that Lp(a), in addition to its known atherogenic characteristics, promotes thrombosis through its effects on platelets and the coagulation cascade^15^. Elevated red blood cell distribution width (RDW) levels are a sign of cytopenia, which is a result of impaired erythropoiesis or erythrocyte degradation^16^. The elevated RDW level indicates the presence of chronic inflammation and heightened oxidative stress^17^. In patients with NVAF, both Lp(a) and RDW are associated with an increased risk of ischemic stroke. Song et al.’s study found a significant association between elevated Lp(a) levels and increased risk of ischemic stroke (OR = 1.23, 95% CI: 1.07–1.41) and systemic embolic events (SEE) (OR = 2.78, 95% CI: 1.78–4.36) in patients with NVAF^18^. Erin et al.^19^ found that in patients with atrial fibrillation, those in the upper tertile of RDW had a doubled risk of developing ischemic stroke (HR = 2.07, 95% CI 1.20–3.57). The objective of this study was to assess the clinical utility of Lp(a) and RDW in conjunction with the CHA_2_DS_2_-VASc-60 score in forecasting acute ischemic stroke among patients with NVAF. The ultimate goal was to establish a benchmark for clinical prevention and treatment.

## Methods

### Study design and participants

The present study included patients who were diagnosed with atrial fibrillation and were consecutively admitted to different clinical departments of the Third Affiliated Hospital of Anhui Medical University throughout the period from April 1, 2020, to April 1, 2023. The exclusion criteria encompassed any of the following: Valvular atrial fibrillation occurring subsequent to mechanical valve replacement, as well as cases of moderate and severe mitral stenosis complicated by AF, are among the medical conditions under consideration. Other relevant conditions include rheumatic heart disease, dilated cardiomyopathy, severe infections, anemia, chronic liver disease, chronic kidney disease, thyroid disorder, cerebral hemorrhage, tumors, and the absence of data pertaining to Lp(a), RDW, and other significant variables. The patients were classified into two distinct groups based on the diagnosis of AIS.

### Assessments of atrial fibrillation and acute ischemic stroke

The diagnosis of atrial fibrillation (AF) in patients was established through the examination of their medical history, 12-lead electrocardiography, or 24-h Holter monitoring^6^. Electrocardiographic characteristics of AF include irregularly irregular R-R intervals (when atrioventricular conduction is not impaired), the absence of distinct repeating P waves, and irregular atrial activations. The diagnosis of NVAF encompasses two subtypes: paroxysmal AF (AF that terminates spontaneously or with intervention within 7 days of onset.) and persistent AF [AF that is continuously sustained beyond 7 days, including episodes terminated by cardioversion (drugs or electrical cardioversion) after ≥ 7 days]. According to the diagnostic criteria for acute ischemic stroke in the International Classification of Diseases, 11th Revision (ICD-11), it includes: acute focal neurological deficit, including complete facial nerve dysfunction; and the presence of symptoms or signs lasting for more than 24 h. Additionally, a causative lesion is identified through brain CT (Computed Tomography) or MRI (Magnetic Resonance Imaging), and individuals with intracerebral hemorrhage are excluded based on imaging^20^.

### Data collection

The hospital’s electronic medical record system was used to retrospectively collect basic patient information, which included age, gender, smoking and alcohol history, comorbidities (such as hypertension, diabetes, coronary heart disease, heart failure, and vascular disease), medication history (including oral antiplatelet, anticoagulant, and antihypertensive drugs), type of atrial fibrillation, CHA_2_DS_2_-VASc score and CHA_2_DS_2_-VASc-60 score. The CHA_2_DS_2_-VASc-60 score is an extension of the CHA_2_DS_2_-VASc score that acknowledges Asian individuals with AF have a heightened risk of stroke at a reduced age threshold. Under this modified scoring system, patients aged 60–64 are awarded 1 point, while those aged 65 years or older are awarded 2 points. Additional data that was collected included post-admission blood pressure (BP), fasting laboratory markers such as triglycerides (TG), total cholesterol (TC), high-density lipoprotein cholesterol (HDL-C), low-density lipoprotein cholesterol (LDL-C), Lp(a), homocysteine (HCY), fasting blood glucose (FBG), uric acid (UA), serum creatinine (Scr), Cystatin C (CysC), glomerular filtration rate (GFR), D-dimer (D-D), neutrophil count (NC), lymphocyte count (LC), platelet count (PLT), hematocrit (HCT), red blood cell distribution width (RDW), as well as the calculation of the neutrophil/lymphocyte ratio (NLR) and platelet/lymphocyte ratio (PLR). The echocardiographic markers that were examined in this study encompassed the left atrial diameter (LAD), left ventricular end-diastolic diameter (LVEDD), and left ventricular ejection percent (LVEF).

### Statistical analysis

The statistical analyses were conducted using SPSS (version 26; IBM Inc., Chicago, IL, USA). Numerical variables following a normal distribution are presented as mean ± standard deviation (SD), and an independent sample t-test is used for between-group comparisons. Non-normally distributed variables are presented as median (interquartile range), and non-parametric tests are used for between-group comparisons. Categorical data is expressed as frequency (percentage), and the chi-square (χ^2^) test is used for between-group comparisons. By employing Spearman correlation analysis, the study investigated the relationship between Lp(a) and RDW levels with the CHA_2_DS_2_-VASc score and CHA_2_DS_2_-VASc-60 score in NVAF patients. This study utilized multivariable logistic regression analysis to include factors that showed statistically significant differences (P < 0.05) between the two groups. The forward selection method was employed to select variables, and adjustments were made for potential confounders. Furthermore, area under the curve (AUC) calculations were performed using receiver operator characteristic (ROC) curves to assess the predictive accuracy of CHA_2_DS_2_-VASc-60 scores, Lp(a), and RDW levels in patients with AIS complicated by NVAF, both independently and in conjunction.

### Ethics declarations

The study protocols received approval from the Ethics Committee of the Third Affiliated Hospital of Anhui Medical University (Approval No. LUNYANPI-2020-54, approval date: March 6, 2020). The study was conducted in accordance with the principles of the Declaration of Helsinki and in compliance with applicable local laws and institutional guidelines. The requirement for individual informed consent was waived by the Ethics Committee of the Third Affiliated Hospital of Anhui Medical University because of the retrospective nature of the study.

## Results

### Baseline characteristics

**Figure 1:**
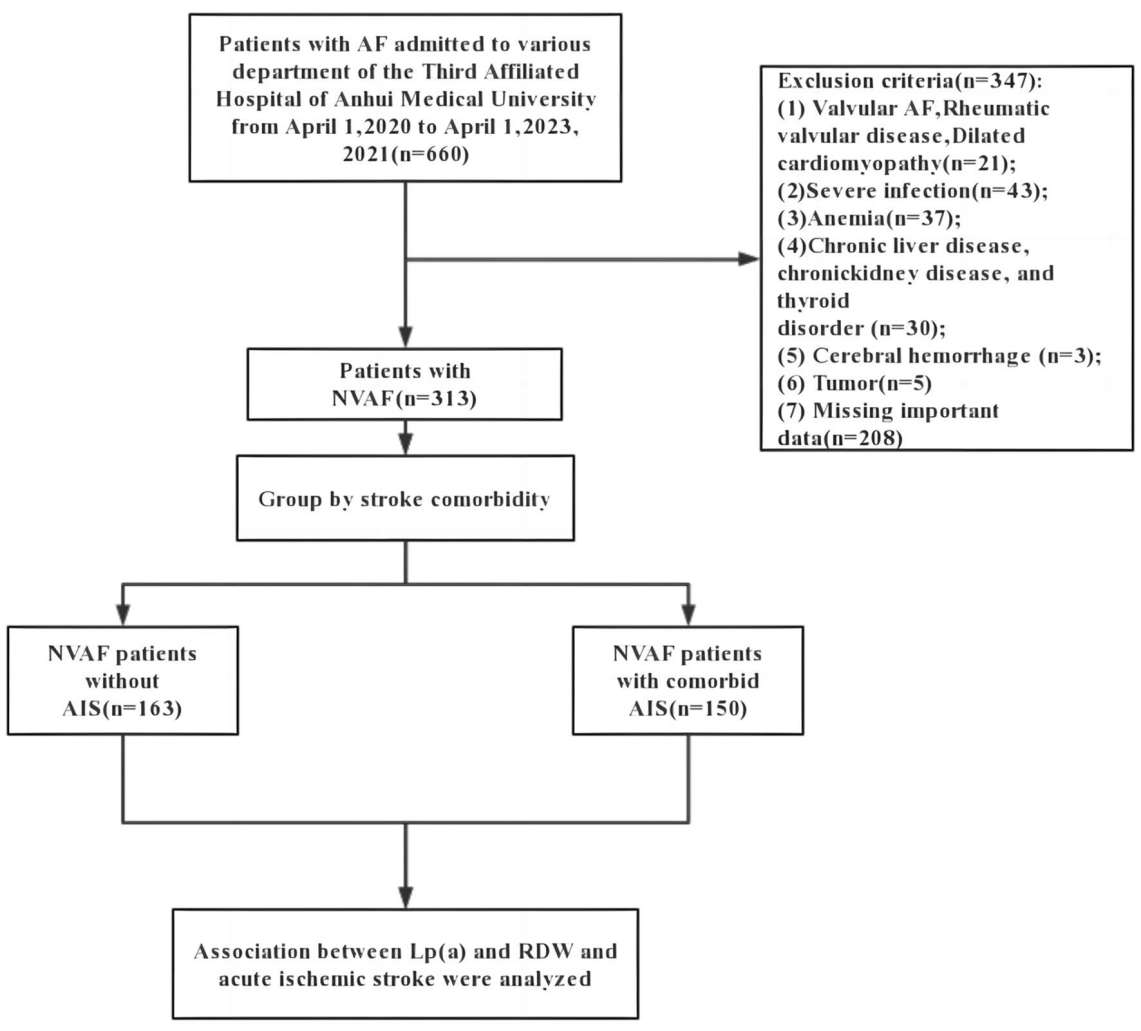
Flow chart for selecting the study population. NVAF, non-valvular atrial fibrillation; AIS, acute ischemic stroke.

Enrolled in this investigation were 313 patients diagnosed with NVAF. The first group consisted of 150 cases of NVAF patients with comorbid AIS, referred to as the AIS group. The second group included 163 cases of NVAF patients without AIS, referred to as the non-AIS group (Fig. 1). Comparison of clinical data between the two groups is shown in Table 1.

**Table 1:** Baseline characteristics of patients with NVAF grouped by AIS.

Characteristics	Non-AIS group (n = 163)	AIS group (n = 150)	*t/χ*^*2*^*/z*	*P*
Age, year	73.28 ± 12.42	78.40 ± 9.10	− 4.186	< 0.001
Male, n%	80 (49.1)	73 (48.7)	0.005	0.942
Smoking, *n*%	41 (25.2)	48 (32.0)	1.799	0.180
Alcohol consumption, n%	34 (20.9)	28 (18.7)	0.236	0.627
Hypertension, n%	105 (64.4)	113 (75.3)	4.403	0.036
Diabetes mellitus, n%	35 (21.5)	43 (28.7)	2.161	0.142
Coronary heart disease, n%	38 (23.3)	38 (25.3)	0.173	0.677
Heart failure, n%	42 (25.8)	46 (30.7)	0.928	0.335
Vascular disease, n%	39 (23.9)	57 (38.0)	7.276	0.007
Antiplatelet drugs, n%	17 (10.4)	39 (26.0)	12.892	< 0.001
Anticoagulant drugs, n%	17 (10.4)	32 (21.3)	7.034	0.008
Antihypertensive drugs, n%	68 (41.7)	94 (62.7)	13.729	< 0.001
Systolic BP, mmHg	130.21 ± 19.06	143.54 ± 23.60	− 5.466	< 0.001
Diastolic BP, mmHg	79.32 ± 14.46	83.21 ± 13.57	− 2.447	0.015
AF type, n%			5.163	0.023
Paroxysmal	85 (52.1)	59 (39.3)		
Persistent	78 (47.9)	91 (60.7)		
CHA_2_DS_2_-VASc score	3.38 ± 1.50	5.39 ± 1.14	− 13.666	< 0.001
CHA_2_DS_2_-VASc-60 score	3.67 ± 1.47	5.68 ± 1.12	− 11.695	< 0.001
TG, mmol/L	1.06 (0.82–1.45)	1.05 (0.83–1.39)	− 0.151	0.880
TC, mmol/L	3.75 ± 0.91	4.19 ± 2.65	− 1.982	0.048
HDL-C, mmol/L	1.14 ± 0.34	1.19 ± 0.31	− 1.474	0.141
LDL-C, mmol/L	2.19 ± 0.82	2.30 ± 0.85	− 1.255	0.210
Lp(a), mg/dL	14.32 (7.96–21.91)	23.98 (13.28–42.22)	− 5.662	< 0.001
HCY, umol/L	15.00 (12.47–20.47)	13.82 (11.11–17.77)	− 2.174	0.030
FBG, ummol/L	5.19 (4.64–6.23)	5.44 (4.70–6.46)	− 0.941	0.346
UA, umol/L	324.48 ± 96.07	331.05 ± 81.59	− 0.649	0.517
Scr, mol/L	82.42 ± 24.64	83.60 ± 18.88	− 0.479	0.632
CysC, mg/L	1.20 (1.05–1.41)	1.23 (1.09–1.40)	− 0.714	0.475
GFR, mL/min	72.87 ± 20.02	68.34 ± 15.18	2.242	0.026
D-D, mg/L	0.39 (0.22–0.76)	0.76 (0.33–1.60)	− 4.763	< 0.001
NC,10^9^/L	3.51 (2.53–4.50)	4.14 (3.34–5.54)	− 4.261	< 0.001
LC,10^9^/L	1.48 ± 0.67	1.51 ± 0.70	− 0.445	0.657
PLT,10^9^/L	183.18 ± 68.95	184.53 ± 52.05	− 0.194	0.846
NLR	2.32 (1.70–3.81)	2.95 (2.11–5.05)	− 2.963	0.003
PLR	117.53 (94.44–171.43)	132.20 (92.12–176.21)	− 0.648	0.517
HCT, %	40.04 ± 4.33	39.69 ± 4.78	0.684	0.494
RDW, %	12.94 ± 0.76	13.67 ± 1.25	− 6.137	< 0.001
LAD, mm	43.28 ± 5.00	44.59 ± 5.09	− 2.296	0.022
LVEDD, mm	49.42 ± 4.25	49.27 ± 4.20	0.328	0.743
LVEF, %	62.84 ± 6.45	61.94 ± 6.28	1.250	0.212

AF, atrial fibrillation; BP, blood pressure; TG, triglycerides; TC, total cholesterol; HDL-C, high-density lipoprotein cholesterol; LDL-C, low-density lipoprotein cholesterol; Lp(a), lipoprotein(a); HCY, homocysteine; FBG, fasting blood glucose; UA, uric acid; Scr, serum creatinine; CysC, Cystatin C; GFR, glomerular filtration rate; D-D, D-dimer; NC, neutrophil count; LC, lymphocyte count; PLT, platelet count; HCT, hematocrit; RDW, red blood cell distribution width; NLR, neutrophil/lymphocyte ratio; PLR, platelet/lymphocyte ratio; LAD, left atrial diameter; LVEDD, left ventricular end-diastolic diameter; LVEF, left ventricular ejection fraction.

### Association of CHA_2_DS_2_-VASc-60 score, Lp(a), and RDW with acute ischemic stroke

A significant difference (P < 0.05) is observed when comparing the CHA_2_DS_2_-VASc-60 score, Lp(a), and RDW of NVAF patients with and without concurrent AIS. The results of the Spearman correlation analysis indicated a positive correlation between the Lp(a) and RDW levels with CHA DS -VASc score and CHA_2_DS_2_-VASc-60 score in patients with NVAF (Table 2).

**Table 2:** Correlation between Lp(a) and RDW with CHA_2_DS_2_-VASc score and CHA_2_DS_2_-VASc-60 score.

Variables	CHA_2_DS_2_ -VASc score		CHA_2_DS_2_-VASc-60 score	
	*r*	*P*	*r*	*P*
Lp(a), mg/dL	0.287	< 0.001	0.312	< 0.001
RDW, %	0.358	< 0.001	0.337	< 0.001

Lp(a), lipoprotein(a); RDW, red blood cell distribution width.

### Risk factors for acute ischemic stroke

Six variables were successfully screened and included in the multivariable logistic regression analysis model as shown in Table 3. Then, we adjusted for age, gender, diabetes, heart failure, vascular diseases, and types of atrial fibrillation. The study showed that the CHA_2_DS_2_-VASc-60 score, Lp(a), and RDW were independent risk factors associated with AIS in non-valvular atrial fibrillation patients (Table 4).

**Table 3:** Multivariable logistic regression analysis of factors associated with AIS.

Index	*β*	*SE*	*Waldχ*^*2*^	*OR* (95%*CI*)	*P*
Hypertension, n%	− 1.157	0.418	7.649	0.314 (0.138–0.714)	0.006
Systolic BP, mmHg	0.016	0.008	3.710	1.016 (1.000–1.033)	0.044
CHA_2_DS_2_-VASc-60 score	1.506	0.193	61.160	4.507 (3.090–6.572)	< 0.001
Lp(a), mg/dL	0.025	0.008	8.923	1.026 (1.009–1.043)	0.003
NC, 10^9^/L	0.169	0.086	3.821	1.184 (1.000–1.401)	0.041
RDW, %	0.388	0.197	3.877	1.474 (1.002–2.169)	0.039

BP, blood pressure; Lp(a), lipoprotein(a); NC, neutrophil count; RDW, red blood cell distribution width.

**Table 4:** Multivariable logistic regression analysis after adjusting for confounders.

Index	*β*	*SE*	*Waldχ*^*2*^	*OR* (95%*CI*)	*P*
Age, year	− 0.012	0.021	0.288	0.989 (0.948–1.031)	0.592
Hypertension, n%	− 1.358	0.453	9.000	0.257 (0.106–0.624)	0.003
Systolic BP, mmHg	0.015	0.009	3.014	1.015 (0.998–1.032)	0.083
CHA_2_DS_2_-VASc-60 score	1.879	0.238	62.553	6.549 (4.110–10.433)	< 0.001
Lp(a), mg/dL	0.023	0.009	6.229	1.023 (1.005–1.041)	0.013
NC, 10^9^/L	0.159	0.096	2.735	1.172 (0.971–1.415)	0.098
RDW, %	0.497	0.219	5.167	1.644 (1.071–2.525)	0.023
Male, n%	1.466	0.411	12.752	4.332 (1.938–9,686)	< 0.001
Diabetes mellitus, n%	− 0.758	0.433	3.065	0.469 (0.201–1.095)	0.080
Heart failure, n%	− 0.640	0.393	2.652	0.527 (0.244–1.139)	0.103
Vascular disease, n%	− 0.161	0.402	0.160	0.851 (0.387–1.872)	0.689
Persistent AF, n%	0.263	0.364	0.521	1.300 (0.637–2.653)	0.470

BP, blood pressure; Lp(a), lipoprotein(a); NC, neutrophil count; RDW, red blood cell distribution width.

### The value of CHA_2_DS_2_-VASc-60 score, Lp(a), and RDW in predicting acute ischemic stroke

**Figure 2:**
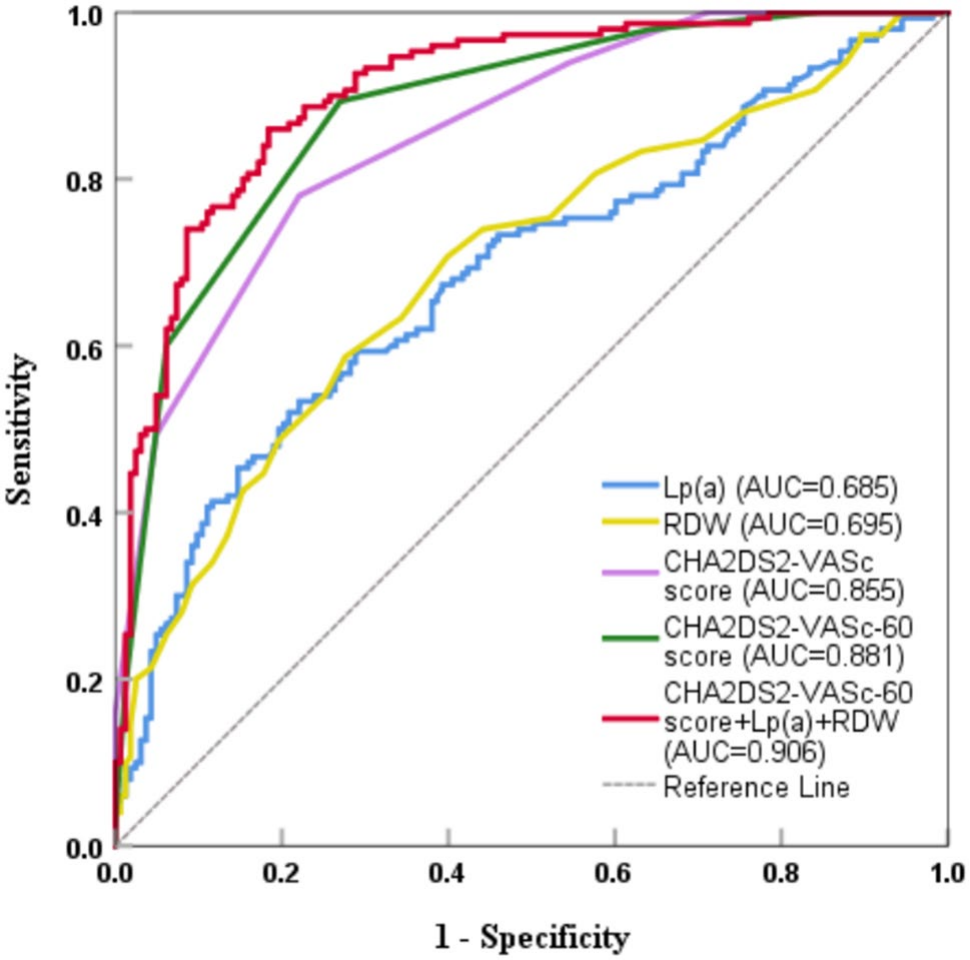
ROC curve analysis of the CHA_2_DS_2_-VASc-60 score, Lp(a), and RDW as predictors of AIS. Lp(a), lipoprotein(a); RDW, red blood cell distribution width.

The CHA_2_DS_2_-VASc-60 score, Lp(a), RDW, and the CHA_2_DS_2_-VASc-60 score in combination with Lp(a) and RDW predicted that NVAF patients with AIS were 0.881, 0.685, 0.695, and 0.906, respectively, according to the ROC curve. The. When Lp(a), RDW, CHA_2_DS_2_-VASc score, and CHA_2_DS_2_-VASc-60 score were used together, the area under the curve was larger than when Lp(a), RDW, CHA_2_DS_2_-VASc score, and CHA_2_DS_2_-VASc-60 score were used separately (all P < 0.01). In addition, the combination of these three factors showed an improvement in specificity for diagnosing acute ischemic stroke in patients with non-valvular atrial fibrillation (NVAF) (0.816 vs. 0. 730) (Table 5, Fig. 2).

**Table 5:** ROC curve analysis of the CHA_2_DS_2_-VASc-60 score, Lp(a), and RDW prediction AIS.

Index	Cutoff value	AUC	95%*CI*	Sensitivity	Specificity	*P*
Lp(a), mg/dL	23.01	0.685	0.626–0.744	0.533	0.779	< 0.001
RDW, %	13.25	0.695	0.637–0.754	0.587	0.724	< 0.001
CHA_2_DS_2_ -VASc score	4.5	0.855	0.814–0.895	0.780	0.779	< 0.001
CHA_2_DS_2_ -VASc-60 score	4.5	0.881	0.844–0.918	0.893	0.730	< 0.001
CHA_2_DS_2_-VASc-60 score combined with Lp(a) and RDW		0.906	0.873–0.939	0.860	0.816	< 0.001

Lp(a), lipoprotein(a); RDW, red blood cell distribution width.

## Discussion

In this study, we found that the AIS group had a higher mean age, hypertension prevalence, proportion of individuals with a history of persistent atrial fibrillation, and CHA_2_DS_2_-VASc score compared to the non-AIS group. These findings are consistent with the investigation conducted by Cha et al.^21^. Therefore, the risk of embolism and ischemic stroke should be comprehensively evaluated for elderly, hypertensive, and persistent atrial fibrillation patients, and anticoagulation or ablation therapy should be actively adopted to improve the prognosis. Moreover, NC, NLR, and D-D levels were also higher in the AIS group than in the non-AIS group, consistent with the results of previous studies by Saliba^22^, Kneihsl^23^, and Guo^24^ (Table 1). This may be attributed to the migration of peripheral blood immune cells dominated by neutrophils to the intracranial injury site through the damaged blood–brain barrier during the onset of ischemic stroke, where these cells secrete various inflammatory mediators and pro-inflammatory factors to exert immune effects^25^. The AIS group exhibited higher values of LAD than the non-AIS group, which is consistent with the findings of Hamatani et al.^26^. This suggests that left atrial enlargement promotes blood stasis, leading to thrombosis and embolism, and LAD may be associated with the duration of atrial fibrillation or the burden of arrhythmias^27^. However, there were no significant differences in gender, diabetes, coronary heart disease, or history of heart failure, which was inconsistent with previous studies. The underlying reasons may be attributed to the limitations of a single-center retrospective study design, a small sample size, and both groups being composed of many elderly individuals with poor general health, more underlying diseases, and comorbidities. Furthermore, the research indicators were potentially influenced by multiple factors, leading to inconsistent findings among studies.

The age of individuals diagnosed with atrial fibrillation is widely regarded as the principal factor in predicting the likelihood of developing an ischemic stroke. Previous studies have demonstrated that Asian individuals aged 50 years and older who have received a diagnosis of AF are more prone to experiencing stroke^28^. Based on prior investigations^29^, it is recommended that the age requirement be adjusted to 55 years in order to accommodate Asian patients more effectively. The results of this investigation suggest that the AIS group exhibited higher values for both the CHA_2_DS_2_-VASc score and the CHA_2_DS_2_-VASc-60 score in comparison to the non-AIS group (Table 1). In addition, the CHA_2_DS_2_-VASc-60 score was identified as an independent risk factor for acute ischemic stroke in patients with NVAF according to the results of the multivariate logistic regression analysis (P < 0.05) (Table 3). Furthermore, it was observed that the AUC for predicting AIS, as assessed by the CHA_2_DS_2_-VASc-60 score, was greater in magnitude compared to the CHA_2_DS_2_-VASc score. According to these results, the CHA_2_DS_2_-VASc-60 score exhibits a higher degree of predictive efficacy in this particular context.

Lp(a) is an independent risk factor for atherosclerotic cardiovascular disease (ASCVD) and stroke, according to many studies^30–32^. It is worth noting that ASCVD significantly increases the likelihood of developing atrial fibrillation. High Lp(a) levels are significantly associated with an increased incidence of ischemic stroke in individuals without atrial fibrillation, as opposed to those with atrial fibrillation, according to findings from the ARIC cohort^33^. Nevertheless, Mohammadi-Shemirani's more recent MR study implies that Lp(a) might also serve as a risk factor for atrial fibrillation^34^. After controlling for potential confounding factors such as CHA_2_DS_2_-VASc scores, an increase of 1 standard deviation (SD) in log-Lp(a) is associated with a 23 percent rise in the risk of infarction in patients with atrial fibrillation. Furthermore, elevated concentrations of Lp(a) are significantly correlated with both infarction and systemic embolism events (SEE)^18^. Kamili and his team’s research found that in non-valvular atrial fibrillation patients with low CHA_2_DS_2_-VASc scores, elevated Lp(a) is an independent risk factor for left atrial thrombus/spontaneous echo contrast^35^. Our investigation revealed a statistically significant increase in Lp(a) concentration within the stroke cohort compared to the control cohort. Furthermore, multivariate logistic regression analysis demonstrated that Lp(a) serves as a risk factor for the onset of AIS in patients diagnosed with NVAF. The pathophysiology underlying Lp(a)-induced AIS in patients with NVAF remains unclear but may involve the following mechanisms. Firstly, Lp(a)-mediated pro-inflammatory effects could impair atrial remodeling and electrical signaling^36^. Phospholipid oxides tend to bind with Lp(a) instead of other LDL particles, and these oxidative phospholipids upregulate IL-8 and monocyte chemotaxis proteins, further promoting inflammation^36,37^. Furthermore, Lp(a) can interfere with the binding of structurally similar molecules such as plasminogen and tissue plasminogen activators to platelet surfaces, resulting in impaired platelet-mediated fibrinolytic reactions^38^. In addition, Lp(a) inactivates tissue factor pathway inhibitors, which may promote thrombosis through exogenous coagulation pathways^39^.

The RDW serves as an indicator of the variability in the volume of peripheral red blood cells. An elevated RDW signifies the existence of erythrocytopenia, which can be attributed to compromised erythropoiesis or erythrocyte degradation. Such conditions are characterized by chronic inflammation and elevated levels of oxidative stress^16^. A substantial body of research has established a strong correlation between RDW and the incidence of cardiovascular events. Adamsson, in particular, identified a positive association between RDW and the progression to atrial fibrillation^40^. In the study conducted by Liu et al.^41^, multivariable logistic regression analysis showed that an elevated RDW was an independent predictive factor for the CHA_2_DS_2_-VASc score (OR: 5.748, P < 0.05). In patients with NVAF, elevated RDW was independently associated with thromboembolic events in a “real-world” retrospective cohort study with a 5.2-year follow-up^42^. In patients with NVAF, the existence of a high RDW (> 13.16%) has also been demonstrated to be correlated with left atrial thrombosis^43^. Results from the current study indicate that the RDW value in the AIS group was higher than that of the non-AIS group, and the multivariate logistic regression analysis further demonstrated that RDW was a risk factor for AIS, consistent with the findings of Wan^44^, Saliba^22^, and others. Possible mechanisms underlying these associations include inflammation-induced alterations in iron metabolism inhibiting erythropoietin and shortening the lifespan of red blood cells^45^. Increased RDW can cause atrial cell damage, leading to atrial structural remodeling and thrombosis^46^. Furthermore, cardiovascular events result in the excitation of the renin-angiotensin system, which, in turn, promotes progenitor erythrocyte activation, leading to increased erythropoiesis and RDW^47^. Based on this simple and readily available test index in clinical practice, the present study explored the predictive value of the CHA_2_DS_2_-VASc-60 score, Lp(a), and RDW for AIS in patients with NVAF. The ROC curve showed that the combined AUC value of CHA_2_DS_2_-VASc-60 score combined with Lp(a) and RDW in predicting AIS in NVAF patients was 0.906, indicating that the combination of the three has a high predictive capacity for AIS in NVAF patients.

### Limitations

This study has several limitations: firstly, it was a single-center retrospective study with a small sample size, so confounding bias may arise. Although we performed multivariate regression analysis to adjust for confounding factors, it is still possible that unmeasured or unmeasurable covariates could play an important role. For example, Karakayali et al.^48^, found that the P-wave index (PWI) obtained from the surface electrocardiogram (ECG) has a certain value in predicting atrial high-rate episodes (AHRE), which is associated with increased risks of AF and thromboembolism^49^. In addition, our study population included a larger number of older individuals with generally poorer health conditions, multiple underlying diseases, and comorbidities, which may affect the generalizability and reproducibility of the results. Secondly, the study only included the first blood sample after hospitalization, and there was no dynamic monitoring of the changing trend of Lp(a) and RDW. Additionally, no follow-up was conducted to further understand the occurrence of stroke and other adverse events in patients in the NVAF group alone. Thirdly, patients with acute ischemic stroke were not classified, and the degree of neurological defects was not scored. In addition, patients with NVAF-comorbid circulatory embolism were not included, and the risk of thrombosis in patients with NVAF was not completely assessed. The next step in this study should include follow-up content to better understand adverse events such as stroke and death in the NVAF group alone. Dynamic changes in Lp(a) and RDW should be recorded, and the predictive value of both for adverse events such as AIS in patients with NVAF should be further verified. Patients with systemic embolism should be included, and stroke patients should be classified and evaluated for the degree of neurological defect to comprehensively assess the value of both parameters for thrombosis risk in patients with NVAF.

## Conclusions

To summarize, patients diagnosed with NVAF who suffered from AIS demonstrated significant increases in the CHA_2_DS_2_-VASc-60 score, Lp (a), and RDW. The study identified these characteristics as independent risk factors for patients with non-valvular atrial fibrillation who experienced an acute ischemic stroke. The simultaneous utilization of these three variables demonstrates a significant potential for prediction in persons diagnosed with NVAF who have encountered an acute ischemic stroke. Therefore, it may function as a supplementary reference indicator for the anticipation of acute ischemic stroke risk in NVAF patients.

## Data Availability

Data is private due to participant confidentiality. The accompanying authors can provide the data sets studied in this work upon reasonable request.
